# A Linear 19-Mer Plant Defensin-Derived Peptide Acts Synergistically with Caspofungin against *Candida albicans* Biofilms

**DOI:** 10.3389/fmicb.2017.02051

**Published:** 2017-10-20

**Authors:** Tanne L. Cools, Caroline Struyfs, Jan W. Drijfhout, Soňa Kucharíková, Celia Lobo Romero, Patrick Van Dijck, Marcelo H. S. Ramada, Carlos Bloch, Bruno P. A. Cammue, Karin Thevissen

**Affiliations:** ^1^Centre of Microbial and Plant Genetics, KU Leuven, Leuven, Belgium; ^2^Department of Plant Systems Biology, VIB, Ghent, Belgium; ^3^Department of Immunohematology and Bloodtransfusion, Leiden University Medical Center, Leiden, Netherlands; ^4^Laboratory of Molecular Cell Biology, KU Leuven, Leuven, Belgium; ^5^Department of Biology, VIB-KU Leuven Center for Microbiology, Leuven, Belgium; ^6^Graduate Program in Genomic Sciences and Biotechnology, Catholic University of Brasilia, Brasilia, Brazil; ^7^Mass Spectrometry Laboratory, Embrapa Genetic Resources and Biotechnology, Brasilia, Brazil

**Keywords:** fungal infections, *Candida*, antimicrobial peptides, biofilms, catheters

## Abstract

Public health problems are associated with device-associated biofilm infections, with *Candida albicans* being the major fungal pathogen. We previously identified potent antibiofilm combination treatment in which the antifungal plant defensin HsAFP1 is co-administered with caspofungin, the preferred antimycotic to treat such infections. In this study, we identified the smallest linear HsAFP1-derived peptide that acts synergistically with caspofungin or anidulafungin against *C. albicans* as HsLin06_18, a 19-mer peptide derived from the C-terminal part of HsAFP1. The [caspofungin + HsLin06_18] combination significantly reduced *in vitro* biofilm formation of *Candida glabrata* and *C. albicans* on catheters, as well as biofilm formation of a caspofungin-resistant *C. albicans* strain. The [caspofungin + HsLin06_18] combination was not cytotoxic and reduced biofilm formation of *C. albicans in vivo* using a subcutaneous rat catheter model, as compared to control treatment. Mode of action research on the [caspofungin + HsLin06_18] combination pointed to caspofungin-facilitated HsLin06_18 internalization and immediate membrane permeabilization. All these findings point to broad-spectrum antibiofilm activity of a combination of HsLin06_18 and caspofungin.

## Introduction

Upon contact, microorganisms have the ability to form biofilms on both biotic and abiotic substrates (Moriarty et al., 2014; Percival et al., 2015). The population of patients suffering from biofilm-related diseases is growing, mainly by the increased use of immunosuppressive therapies and medical devices, such as catheters and implants, which can act as a substrate for biofilm formation (Clark and Hajjeh, 2002; Cauda, 2009; Kaur et al., 2015). Such device-mediated infections can result in a systemic infection. To ensure good recovery of the patients, removal of the device is most often required (Walsh and Rex, 2002; Lebeaux et al., 2014).

*Candida* species are the most common fungal biofilm-associated pathogens, with *Candida albicans* being the most prevalent (Nobile and Johnson, 2015). However, infections caused by non-*albicans Candida* species are increasingly observed in the clinic (Deorukhkar et al., 2014; Udayalaxmi et al., 2014; Muadcheingka and Tantivitayakul, 2015; Gong et al., 2016). Fungal biofilms are characterized by increased tolerance to various antifungal agents (antimycotics) and hence, are very difficult to eradicate. Only few conventional antimycotics, such as echinocandins (e.g., caspo-, mica-, and anidulafungin) and liposomal formulations of amphotericin B, can be used to treat such fungal biofilm-related infections (Fiori et al., 2011; Uppuluri et al., 2011). However, there are drawbacks associated with these antimycotics, including their costs, toxicity and/or resistance occurrence (De Cremer et al., 2015; Tsui et al., 2016). Therefore, there is a need for the identification and characterization of novel antifungal agents, which are also effective against fungal biofilms. An alternative strategy to combat fungal biofilms is combination therapy in which two compounds with different mode of actions are combined (De Cremer et al., 2015). Many advantages are associated with these combination therapies over mono therapies, such as (i) broadening spectrum of drug activity, (ii) synergy, (iii) lowered effective doses, (iv) reduced risk of resistance occurrence, and (v) more rapid antifungal effects (Mukherjee et al., 2005; Fohrer et al., 2006; Bink et al., 2011).

As postulated by the World Health Organization, plants are considered to be excellent sources for development of a wide diversity of drugs (Sardi et al., 2013). In this context, the family of plant defensins is considered as a rich source of antifungal peptides, characterized by broad-spectrum activity against plant and human fungal pathogens (Osborn et al., 1995; Almeida et al., 2000; Thevissen et al., 2007; Mello et al., 2011). Some of these peptides display potent activity against biofilms of *C. albicans* (Vriens et al., 2015a,b) and are in general non-toxic to human cells (Thevissen et al., 2007; Vriens et al., 2015b). They have a low *in vitro* frequency of resistance occurrence (Thevissen et al., 2007) and possess good *in vivo* efficacy (Tavares et al., 2008). In this study we focussed on the plant defensin HsAFP1 isolated from *Heuchera sanguinea*. Besides being characterized by antibiofilm activity, HsAFP1 has the ability to act synergistically with amphotericin B and caspofungin against planktonic and biofilm cultures, with HsLin06, a linear peptide spanning the C-terminal part of HsAFP1, being responsible for both activities (Vriens et al., 2015b). The purpose of the current study was to further investigate the synergistic potential of HsLin06 on the caspofungin's antibiofilm activity in view of clinical applications. Therefore, we first delineated the minimal amino acid sequence of HsLin06 that is responsible for this synergistic activity. To this end, we assessed the activity of a series of truncated HsLin06-based peptide variants and the smallest active HsLin06-variant (HsLin06_18) was selected for further experiments. Furthermore, after confirming that this peptide was not cytotoxic to human cells, its efficacy in combination with caspofungin was assessed against biofilms formed on catheters in both *in vitro* and *in vivo* setups. Moreover, we investigated the synergistic potential of HsLin06_18 with other echinocandins used in the clinic as well as the activity spectrum of the [caspofungin-HsLin06_18] combination against different *Candida* species including the caspofungin resistant strains. Finally, we further unraveled the mode of action of the [caspofungin-peptide] combination in more detail, including a structure-activity relationship study, confocal microscopy-based internalization and membrane permeabilization assays.

## Materials and methods

### Strains and reagents

Following *Candida* strains were used in this study: *C. albicans* SC5314 WT (Fonzi and Irwin, 1993), *C. albicans* caspofungin-resistant mutant M177 (Garcia-Effron et al., 2009), *Candida dubliniensis* NCPF 3949 (Sullivan et al., 1995), *Candida glabrata* BG2 (Kaur et al., 2007), and *Candida krusei* IHEM 6104 (Belgian Coordinated Collections of Microorganisms, Brussels, Belgium). HsLin06_18 cytotoxicity tests were performed on HepG2 (Knowles et al., 1980), purchased from ATCC (HB-8065; Rockville, USA).

*Candida albicans* was grown overnight in YPD (1% yeast extract, 2% peptone, and 2% glucose), with all compounds purchased from LabM (UK). All biofilm experiments were performed in RPMI-1640 medium (Roswell Park Memorial Institute-1640 medium; pH7) with L-glutamine and without sodium bicarbonate (purchased from Sigma Aldrich, St Louis, USA), buffered with MOPS (MP Biomedicals Europe, France). Caspofungin (cancidas), micafungin (mycamine) and anidulafungin (ecalta) were purchased from Merck (UK), Astellas Pharma Europe (The Netherlands) and Pfizer (UK), respectively. 16-24-mer HsLin06-derived linear peptides (HsLins) were synthesized, according to the Fmoc protocol, and purified as described previously (Goblyos et al., 2013). To label HsLin06_18 with the fluorescent dye FITC, the resin-bound peptide was elongated with Fmoc-beta-alanine. After removal of the Fmoc protection, the N-terminus of the peptide was reacted with FITC to incorporate the label. TFA-mediated cleavage of the peptide from the resin and removal of the side chain protecting groups, as well as reversed phase HPLC-purification of the labeled peptide was performed as described previously (Goblyos et al., 2013). For the *in vitro* catheter experiments, bovine serum (Sigma Aldrich, St Louis, USA) was used. The dye propidium iodide, used in the flow cytometry and microscopy experiments, was purchased from Sigma Aldrich (St Louis, USA).

### *In vitro* biofilm inhibition assay in microtiter plates

The biofilm inhibition assay in polystyrene microtiter plates was performed as described previously (Vriens et al., 2015b), except for the quantification method. Briefly, *Candida* cells were incubated at OD_600 nm_ = 0.1 in RPMI in round-bottomed microtiter plates (TPP, Tradingen, Switzerland). After 1 h of adhesion at 37°C, biofilms were washed and subsequently incubated for 24 h with fresh RPMI containing HsLin and/or an echinocandin (Vriens et al., 2015b). All *C. albicans* biofilms were quantified by incubating the treated biofilms for 1 h at 37°C with 1/100 dilution of the metabolic dye CTB (Promega, Madison, USA) in PBS (Vriens et al., 2015b), except for the *C. albicans* and non-*albicans Candida* species biofilms in **Figure 5**, which were quantified using XTT. In the latter, 110 μL of a XTT solution (0.25 mg/mL in PBS, 1 μM menadione; Sigma Aldrich, St Louis, USA) was added to the biofilms. After 1 h at 37°C, the absorption (OD_490 nm_) of 100 μL of the converted XTT solution was measured. Next, the Biofilm Inhibitory Concentration 50 (BIC_50_) value, i.e., the concentration of the compound required for 50% biofilm formation inhibition, compared to the control treatment (0.5% DSMO), was determined. Checkerboard assays were performed for [echinocandin + HsLin] combinations (Vriens et al., 2015b), and the fold change of echinocandin's BIC_50_ was determined, i.e., the ratio of echinocandin's BIC_50_ in the absence/presence of the peptide. To test synergistic interactions between two agents against biofilm formation, the Fractional Inhibitory Concentration Index (FICI) was calculated (Odds, 2003; Delattin et al., 2014).

### Fungicidal activity assay

Exponentially growing *C. albicans* SC5314 WT cells were incubated at OD_600 nm_ = 1 in RPMI with caspofungin (0.01 μM), HsLin06_18-FITC (4.6 μM) or its combination for 10–40 min at 37°C and 200 rpm. Both at the start and at all measuring points, surviving yeast cells was determined via plating assays. In a plating assay, 10-fold dilution series of yeast cells in PBS were prepared, after which 100 μL was plated on YPD plates. After 1 day of incubation at 37°C, the number of colony forming units (CFU) was counted and cell death was calculated relative to time zero (t0). MFC50 values, indicating the minimum fungicidal concentration resulting in 50% cell death, were determined. In parallel with the plating assay, confocal microscopic or flow cytometric analysis were performed on the samples.

### Confocal microscopy

HsLin06_18 localization studies were performed on *C. albicans* SC5314 WT cells treated with the [caspofungin + HsLin06_18] combination or its corresponding mono treatments, both on planktonic and biofilm cultures. In parallel, membrane permeabilization was investigated via the fluorescent dye propidium iodide (PI). For the biofilm setup, biofilms were grown and treated for 24 h with caspofungin (0.625 μM), HsLin06_18-FITC (0.5 μM) or its combination, in microtiter plates, as described in the materials and methods section “*in vitro* biofilm inhibition assay in microtiter plates.” After a PBS-washing step, these biofilms were incubated for 20 min at 20°C with 100 μL PI solution (30 μg/mL PI in PBS). Next, the biofilms were resuspended by up- and down pipetting, after which they were visualized by confocal microscopy with the FluoView FV1000 confocal microscope (Olympus IX81) and its software. We used a 60x magnification objective and 3.5x computer zoom. The 488 nm laser line of the Argon laser was used for visualization of BODIPY and the 559 nm laser for PI. For the planktonic setup, exponentially growing cells were incubated at OD_600 nm_ = 1 in RPMI with caspofungin (0.01 μM), HsLin06_18-FITC (4.6 μM) or its combination (as described in the materials and methods section “fungicidal activity assay”), together with PI (2 μg/mL) for 40 min. Next, cells were pelleted and concentrated 10-times in PBS, after which they were visualized by confocal microscopy.

### Flow cytometry

Membrane permeabilization and HsLin06_18 internalization were determined via flow cytometric analysis on planktonic *C. albicans* SC5314 WT cells. Exponentially growing cells were exposed to caspofungin (0.01 μM), HsLin06_18-FITC (4.6 μM) or its combination (as described in the material and methods section “fungicidal activity assay”), and to propidium iodide (PI) (2 μg/mL), after which they were subjected to flow cytometry on a BD Influx™ cell sorter. Per treatment, 10,000 cells were monitored for fluorescence at 530/40 nm (FL2_λ_ex_ = 488 nm) and 610/20 nm (FL11_ λ_ex_ = 561 nm) for the detection of HsLin06_18-FITC internalization (FITC+) and membrane permeabilization (PI+), respectively. Data from cells treated with 4.6 μM HsLin06_18-FITC were used as background signal.

### *In vitro* biofilm inhibition assay on catheters

Polyurethane catheters, overnight incubated in bovine serum, were infected with *C. albicans* or *C. glabrata* cells (5 × 10^4^ cells/mL) in RPMI medium (Kucharikova et al., 2015). After an adhesion phase (90 min at 37°C), these catheters were washed and subsequently treated with caspofungin (0.4 μM), HsLin06_18 (0.5 μM), its combination or control treatment (0.5% DMSO) for 24 h, after which the number of cells per individual biofilm was determined by CFU (Kucharikova et al., 2015). All experiments were performed in quadruplicate with at least three biological repeats.

### HsLin06_18 cytotoxicity assays

Drug cytotoxicity testing was performed as described previously (Vriens et al., 2015b). Briefly, human hepatoma (HepG2) cells were treated for 24 h with caspofungin, HsLin06_18, its combination or control treatment (1% DMSO). Cell viability was determined by MTT staining (Roche Diagnostics, Germany) and expressed relative to the control treatment. All experiments were performed in quadruplicate with at least three biological repeats.

### *In vivo* biofilm inhibition assay

All animal experiments were in accordance with the KU Leuven animal care guidelines and approved by the Ethical Committee of the KU Leuven (P069/2016). *In vivo C. albicans* biofilms were formed inside catheter pieces in immunosuppressed female Sprague–Dawley rats (200 g) as described previously (Ricicova et al., 2010). Briefly, serum pre-incubated catheters were infected with *C. albicans* cells (5 × 10^4^ cells/mL) prepared in RPMI medium during the adhesion period (90 min at 37°C). After a washing step, 9 catheter pieces were implanted into the back area of one rat. Inhibition of *in vivo C. albicans* biofilm formation was initiated immediately after the implant. Caspofungin, HsLin06_18, the combination or the control (0.9% NaCl and 0.2% DMSO) treatment were administered intravenously or subcutaneously (in the surroundings of the catheters), once daily for 7 days. Catheters were explanted from euthanized animals and the number of cells per individual biofilm was determined by CFU (Ricicova et al., 2010). *In vivo* experiments were performed once (in total three animals/group).

### Data analysis

Data were analyzed with GraphPad Prism 6. Significant differences were determined on means ± SEM with *P* < 0.05 as considered statistically significant. For the *in vitro* biofilm inhibition assay in microtiter plates, significance between the caspofungin and the [caspofungin + HsLin] combination treatment was determined via unpaired student *t-test*s on BIC_50_ ± SEM values. For the kinetic experiments, the first time point at which HsLin06_18-FITC internalization and/or membrane permeabilization are significantly different from t0 were determined via two-way ANOVA followed by Dunnett multiple comparison for each treatment. For the flow cytometric experiments on WT and *fks1* mutant cells, significant differences in the size of the subpopulations between both strains, were determined via two-way ANOVA followed by Sidak multiple comparison for each treatment. For the *in vitro* and *in vivo* [caspofungin + HsLin06_18] combination test on catheters, CFU's and 95% confidence intervals (CI) values were analyzed for all treatments via one-way ANOVA followed by Tukey multiple comparison, while for the *in vitro* and *in vivo* caspofungin test on catheters, CFU's ± SEM values of all caspofungin concentrations were analyzed relative to the control treatment via one-way ANOVA followed by Dunnett multiple comparison. For the cytotoxicity test, HepG2 cell viability was analyzed for all tested concentrations via one-way ANOVA followed by Tukey multiple comparison.

## Results

### Different regions in HsLin06 are responsible for antibiofilm and synergistic activity with caspofungin

In previous studies we showed that the antifungal plant defensin HsAFP1 is characterized by antibiofilm activity and by synergistic activity with caspofungin against *C. albicans* biofilms (Vriens et al., 2015b). By scanning the entire HsAFP1 sequence with six 24-mer linear peptides, the C-terminal part of HsAFP1 (corresponding to HsLin06) was identified as the region important for HsAFP1's antibiofilm activity and synergy with caspofungin. Here, we tested a series of 44 HsLin06-variants (HsLin06_01-44) with N- or C-terminal truncations of the HsLin06 amino acid sequence, ranging from 16 to 23 amino acids (Table 1). Their antibiofilm activity in microtiter plates was evaluated by determining BIC_50_ values, i.e., the minimal peptide concentration resulting in inhibition of 50% biofilm formation. Their potential to increase the antibiofilm activity of caspofungin against *C. albicans* biofilms was evaluated by comparing the reduction of the BIC_50_ of caspofungin in the presence and absence of the HsLin06-variant, represented as fold change values (Table 1).

**Table 1:** *Candida albicans* biofilm formation inhibition of HsLin06-variants alone or in combination with caspofungin, using microtiter plates^e^.

			**HsLin alone**	**Caspofungin + HsLin**
**Name**	**#aa**^a^	**Sequence**^b^	**BIC_50_^c^ HsLin (μM)**	**Fold change**^d^
HsLin06	24	EHFAYGGAXHYQFPSVKXFXKRQX	0.53	2,56
HsLin06_01	23	HFAYGGAXHYQFPSVKXFXKRQX	0.71	4.98
HsLin06_02	23	EHFAYGGAXHYQFPSVKXFXKRQ	0.56	10.62
HsLin06_03	22	FAYGGAXHYQFPSVKXFXKRQX	0.56	2.52
HsLin06_04	22	HFAYGGAXHYQFPSVKXFXKRQ	0.38	1.01
HsLin06_05	22	EHFAYGGAXHYQFPSVKXFXKR	0.46	2.47
HsLin06_06	21	AYGGAXHYQFPSVKXFXKRQX	1.16	11.14
HsLin06_07	21	FAYGGAXHYQFPSVKXFXKRQ	0.50	1.16
HsLin06_08	21	HFAYGGAXHYQFPSVKXFXKR	0.48	1.22
HsLin06_09	21	EHFAYGGAXHYQFPSVKXFXK	>2	7.99
HsLin06_10	20	YGGAXHYQFPSVKXFXKRQX	0.84	12.49
HsLin06_11	20	AYGGAXHYQFPSVKXFXKRQ	0.78	1.09
HsLin06_12	20	FAYGGAXHYQFPSVKXFXKR	0.71	1.17
HsLin06_13	20	HFAYGGAXHYQFPSVKXFXK	>2	12.15
HsLin06_14	20	EHFAYGGAXHYQFPSVKXFX	>2	3.65
HsLin06_15	19	GGAXHYQFPSVKXFXKRQX	0.90	1.04
HsLin06_16	19	YGGAXHYQFPSVKXFXKRQ	1.03	1.72
HsLin06_17	19	AYGGAXHYQFPSVKXFXKR	>2	1.49
**HsLin06_18**	**19**	**FAYGGAXHYQFPSVKXFXK**	>**2**	**10.42**
HsLin06_19	19	HFAYGGAXHYQFPSVKXFX	>2	3.15
HsLin06_20	19	EHFAYGGAXHYQFPSVKXF	>2	1.44
HsLin06_21	18	GAXHYQFPSVKXFXKRQX	>2	1.49
HsLin06_22	18	GGAXHYQFPSVKXFXKRQ	0.63	4.37
HsLin06_23	18	YGGAXHYQFPSVKXFXKR	1.11	2.06
HsLin06_24	18	AYGGAXHYQFPSVKXFXK	>2	5.36
HsLin06_25	18	FAYGGAXHYQFPSVKXFX	>2	5.83
HsLin06_26	18	HFAYGGAXHYQFPSVKXF	1.52	5.31
HsLin06_27	18	EHFAYGGAXHYQFPSVKX	>2	2.01
HsLin06_28	17	AXHYQFPSVKXFXKRQX	2.01	1.55
HsLin06_29	17	GAXHYQFPSVKXFXKRQ	0.95	1.73
HsLin06_30	17	GGAXHYQFPSVKXFXKR	0.80	2.58
HsLin06_31	17	YGGAXHYQFPSVKXFXK	>2	6.96
HsLin06_32	17	AYGGAXHYQFPSVKXFX	>2	3.10
HsLin06_33	17	FAYGGAXHYQFPSVKXF	>2	7.22
HsLin06_34	17	HFAYGGAXHYQFPSVKX	>2	2.72
HsLin06_35	17	EHFAYGGAXHYQFPSVK	>2	3.34
HsLin06_36	16	XHYQFPSVKXFXKRQX	1.01	1.92
HsLin06_37	16	AXHYQFPSVKXFXKRQ	1.52	1.50
HsLin06_38	16	GAXHYQFPSVKXFXKR	0.66	2.21
HsLin06_39	16	GGAXHYQFPSVKXFXK	>2	3.99
HsLin06_40	16	YGGAXHYQFPSVKXFX	>2	3.79
HsLin06_41	16	AYGGAXHYQFPSVKXF	>2	2.85
HsLin06_42	16	FAYGGAXHYQFPSVKX	>2	2.96
HsLin06_43	16	HFAYGGAXHYQFPSVK	>2	2.96
HsLin06_44	16	EHFAYGGAXHYQFPSV	>2	2.03

a *#aa, number of amino acids*.

b *Sequence, amino acid sequence of the HsLin06-variants (full HsAFP1 sequence showed in Figure 1) in which X = α-ABA*.

c *BIC_50_ values, i.e., the minimum inhibitory concentration resulting in 50% biofilm inhibition compared to the control treatment*.

d *Fold change values, i.e., the reduction of the BIC_50_ of caspofungin by co-incubation with HsLin06 or HsLin06-variants*.

^e^HsLin06-variants with a similar BIC_50_ as HsLin06 [0.5 ^*^ BIC_50_(HsLin06) < BIC_50_(HsLin) < 2 ^*^ BIC_50_(HsLin06)] are marked in light gray. Fold change values larger than 10 (i.e., 10-fold improvement of caspofungin's antibiofilm activity) are marked in dark gray. HsLin06_18, the smallest HsLin06-variant with fold change values >10, is marked in bold. Data are means for n = 3 independent experiments.

We found that none of the tested HsLin06-variants was characterized by significantly better antibiofilm activity than HsLin06. Equally potent antibiofilm activity was found for 17 HsLin06-variants (light gray in Table 1), whereas 27 HsLin06-variants performed worse than HsLin06. In addition, we found that five HsLin06-variants, namely HsLin06_02/06/10/13 and HsLin06_18 (dark gray in Table 1), reduced the BIC_50_ of caspofungin by approximately 10-fold irrespective whether they displayed potent or less potent antibiofilm activity on their own (quantified by their BIC_50_ values), with HsLin06_18 being the shortest active peptide (containing 19 amino acids). Therefore, HsLin06_18 was selected as the best peptide that increased caspofungin's antibiofilm activity.

To test whether HsLin06_18 acts synergistically with caspofungin against biofilms, corresponding FICI values were determined (Supplementary Table S1). For 0.5 μM and 0.25 μM HsLin06_18, FICI values were lower than 0.5, pointing to its synergistic interaction with caspofungin against *C. albicans* biofilms.

Based on all the data in Table 1, we delineated the core sequence of HsAFP1 for antibiofilm activity as GACHYQFPSVKCFCKR (Figure 1, boxed in red) and for increased antibiofilm activity in combination with caspofungin as AYGGACHYQFPSVKCFC (Figure 1, boxed in blue), with cysteines being replaced by X = α-ABA in all HsLins. Both core sequences are only partially overlapping (GACHYQFPSVKCFC), suggesting that both activities contained in HsLin06 are not fully linked. However, both regions are still situated in the γ-core region, which is a region that is important for the antifungal activity of many plant defensins (De Samblanx et al., 1996; Sagaram et al., 2011).

**Figure 1:**
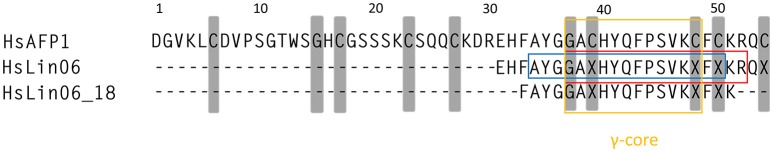
HsAFP1 core regions responsible for prevention of *Candida albicans* biofilm formation (red box) and synergy with caspofungin (blue box), identified by screening HsLin06-variants (Table 1) for both activities. Both regions are situated within the γ-core of plant defensins (yellow box). Gray regions represent conserved amino acids among plant defensins.

### Structure-activity relationship study of HsLin06_18

To get more insight into the structure-activity relationship of HsLin06_18, we performed a full alanine scan of HsLin06_18, resulting in 19 HsLin06_18-variants (HsLin06_18_01-19), which were subsequently tested for their antibiofilm activity in combination with caspofungin (Table 2). None of the amino acid replacements in HsLin06_18 resulted in an abolishment of its activity in combination with caspofungin. Moreover, the fold change values of all peptides were in the same range, indicating that the exact amino acid sequence of HsLin06_18 seems not that important.

**Table 2:** *Candida albicans* biofilm formation inhibition of HsLin06_18-variants in combination with caspofungin, in an *in vitro* microtiter plate assay^a^.

		**Caspofungin + HsLin**
**Name**	**Sequence**	**Fold change^b^**
HsLin06_18	FAYGGAXHYQFPSVKXFXK	7.49
HsLin06_18_01	**A**AYGGAXHYQFPSVKXFXK	5.91
HsLin06_18_02	F**G**YGGAXHYQFPSVKXFXK	8.99
HsLin06_18_03	FA**A**GGAXHYQFPSVKXFXK	6.63
HsLin06_18_04	FAY**A**GAXHYQFPSVKXFXK	7.66
HsLin06_18_05	FAYG**A**AXHYQFPSVKXFXK	5.54
HsLin06_18_06	FAYGG**G**XHYQFPSVKXFXK	7.70
HsLin06_18_07	FAYGGA**A**HYQFPSVKXFXK	7.65
HsLin06_18_08	FAYGGAX**A**YQFPSVKXFXK	5.03
HsLin06_18_09	FAYGGAXH**A**QFPSVKXFXK	5.66
HsLin06_18_10	FAYGGAXHY**A**FPSVKXFXK	8.18
HsLin06_18_11	FAYGGAXHYQ**A**PSVKXFXK	4.90
HsLin06_18_12	FAYGGAXHYQF**A**SVKXFXK	4.75
HsLin06_18_13	FAYGGAXHYQFPAVKXFXK	11.72
HsLin06_18_14	FAYGGAXHYQFPS**A**KXFXK	5.89
HsLin06_18_15	FAYGGAXHYQFPSV**A**XFXK	7.07
HsLin06_18_16	FAYGGAXHYQFPSVK**A**FXK	6.17
HsLin06_18_17	FAYGGAXHYQFPSVKX**A**XK	7.22
HsLin06_18_18	FAYGGAXHYQFPSVKXF**A**K	6.31
HsLin06_18_19	FAYGGAXHYQFPSVKXFX**A**	6.65
HsLin06_18_20	FAYGGA**C**HYQFPSVKXF**C**K	1.97
HsLin06_18_21	FAYGGAXHYQFPSVK**C**F**C**K	NS^c^
HsLin06_18_22	FAYGGA**C**HYQFPSVK**C**FXK	1.77

a *Amino acid replacements in HsLin06_18_01-19, as compared to HsLin06_18, are marked in bold. Amino acid replacements resulting in elevated activity are boxed in gray. Cysteines involved in disulphide bridges for cyclization are underlined for HsLin06_18_20-22. Data are means for n ≥ 2 independent experiment*.

b *Fold change values represent the reduction of the BIC_50_, i.e., the minimum inhibitory concentration resulting in 50% biofilm inhibition compared to the control treatment, of caspofungin by co-incubation with HsLin06_18 or HsLin06_18-variants*.

c *NS, not synthesized*.

### Effect of HsLin06_18 on the antibiofilm activity of other echinocandins

**Figure 2:**
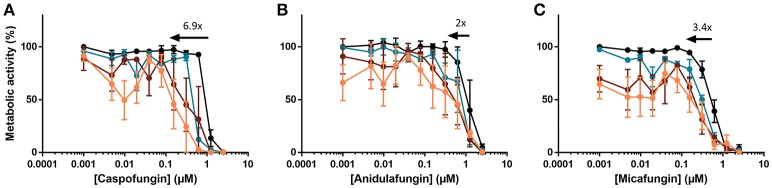
The [echinocandin + HsLin06_18] combination affects *Candida albicans* biofilms, determined in an *in vitro* microtiter plate assay. Dose-response curves of the echinocandin [caspofungin **(A)**, anidulafungin **(B)** and micafungin **(C)**] are presented with different colors for different HsLin06_18 concentrations; orange, 1.25 μM, red, 0.313 μM, blue, 0.156 μM and black 0 μM HsLin06_18. Black arrows represent the HsLin06_18's potentiation effect on echinocandin's action, i.e., the shift of the echinocandin concentration needed for 50% reduction of metabolic activity between the echinocandin and the [echinocandin + 1.25 μM HsLin06_18] treatment. Data are means ± SEM for *n* = 3 independent experiments.

Besides caspofungin, other echinocandins, such as micafungin and anidulafungin are used in the clinic to treat patients suffering from invasive fungal infections (Pfaller et al., 2008). Therefore, we examined the effect of HsLin06_18 on anidulafungin's and micafungin's antibiofilm activity. We found that HsLin06_18 significantly increased the antibiofilm activity of anidulafungin (Figure 2B and Supplementary Table S2). Although increased antibiofilm activity of micafungin in combination with HsLin06_18 was observed (Figure 2C), this effect was not significant, as the BIC_50_ value of micafungin was not significantly decreased in the presence of HsLin06_18 (Supplementary Table S2). Therefore, the [caspofungin + HsLin06_18] combination was selected as the best combination to inhibit *C. albicans* biofilm formation (Figure 2A) and used for further experiments in this study.

### Sub-inhibitory caspofungin doses facilitate HsLin06_18 internalization

**Figure 3:**
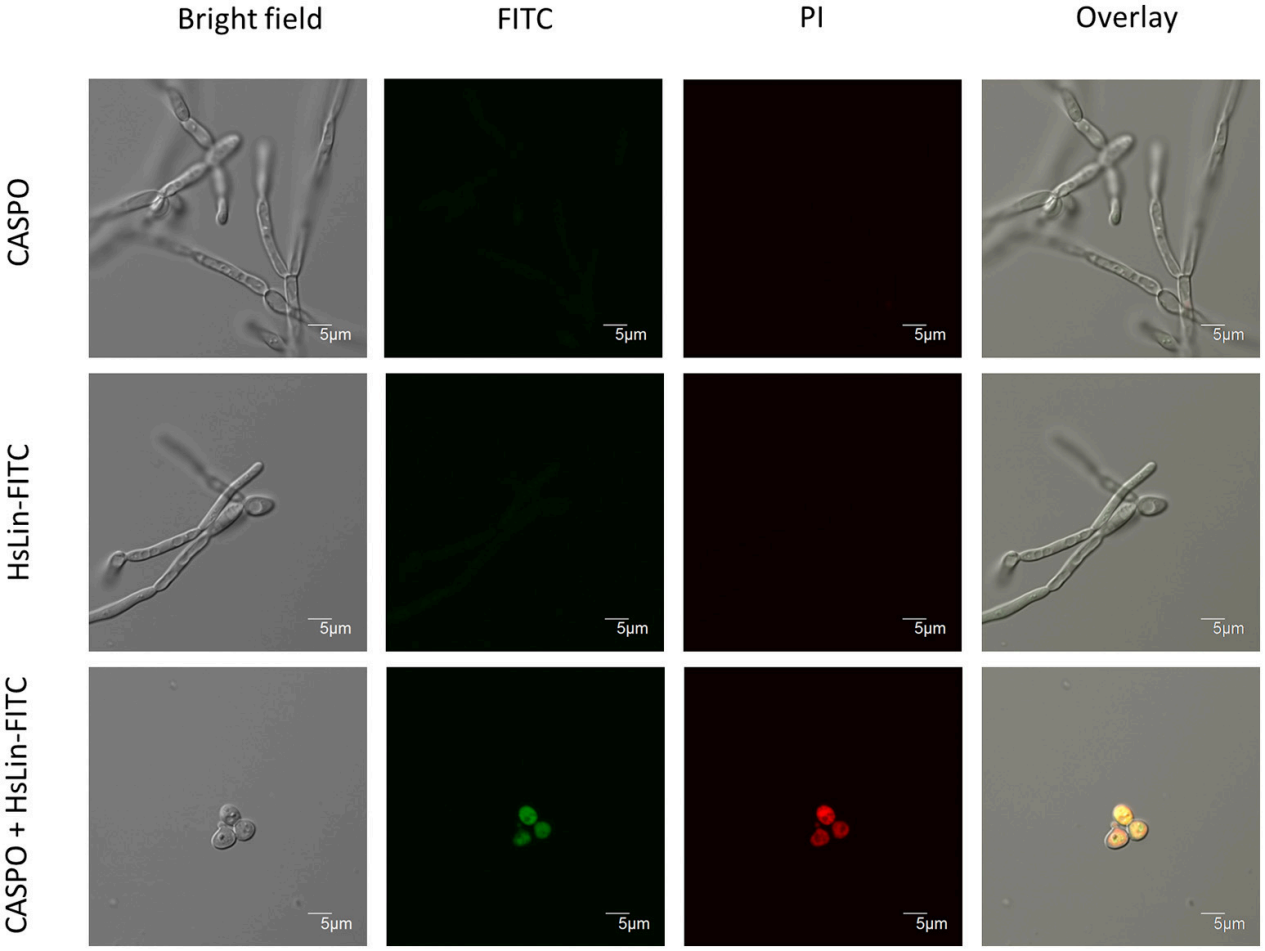
HsLin06_18-FITC uptake and membrane permeabilization in *Candida albicans* biofilms treated with the [caspofungin + HsLin06_18-FITC] combination. Confocal microscope images of 24 h treated *C. albicans* biofilms with 0.625 μM caspofungin (CASPO) and/or 0.5 μM HsLin06_18-FITC and 30 μg/mL propidium iode (PI). Bar: 5 μm.

To get more insight in the mode of action of the [caspofungin + HsLin06_18] combination, we investigated peptide internalization and membrane permeabilization of treated *C. albicans* cells. Our recent results show that HsAFP1-derived peptides, in contrast to native HsAFP1, are not internalized by yeast cells nor possess antifungal activity (Cools et al., submitted). Therefore, we investigated whether caspofungin increased HsLin06_18's internalization in *C. albicans* biofilm cells using N-terminally FITC-labeled HsLin06_18, which is equally potent than unlabelled HsLin06_18 (data not shown). We treated *C. albicans* biofilms with HsLin06_18-FITC in the absence or presence of caspofungin and visualized the biofilm cells with confocal microscopy. In addition, to correlate HsLin06_18-FITC internalization with yeast cell death, the biofilm cells were co-stained with the red fluorescent dye propidium iodide (PI), staining cells with permeabilized membranes (and thus dead). Only *C. albicans* biofilm cells that received the [caspofungin + HsLin06_18-FITC] combination were characterized by green and red fluorescence indicating that they internalized the peptide and had compromised membranes (Figure 3). Biofilm-specific hyphal cells were apparent in the biofilms treated with sub-inhibitory caspofungin or HsLin06_18-FITC doses, while no hyphal cells were found in the biofilms treated with the [caspofungin + HsLin06_18-FITC] combination. Next we examined whether the [caspofungin + HsLin06_18-FITC] combination is also active against planktonic *C. albicans* cells. First, we determined the minimal doses of caspofungin and HsLin06_18-FITC that result in killing of exponentially grown *C. albicans* as [0.01 μM caspofungin + 4.6 μM HsLin06_18-FITC]. In line with the biofilm results, HsLin06_18-FITC internalization and membrane permeabilization occurred only in planktonic *C. albicans* cells that were treated with the [0.01 μM caspofungin + 4.6 μM HsLin06_18-FITC] combination and not when treated with caspofungin or HsLin06_18-FITC alone.

**Figure 4:**
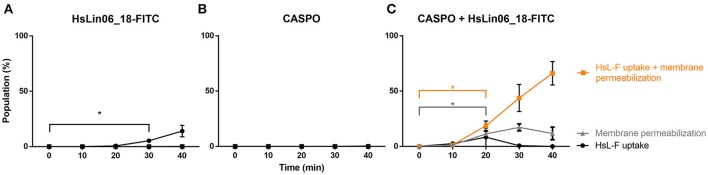
Kinetics of HsLin06_18-FITC uptake and membrane permeabilization in planktonic *Candida albicans* cells treated with **(A)** 4.6 μM HsLin06_18-FITC (HsL-F), **(B)** 0.01 μM caspofungin (CASPO) or **(C)** its combination as well as 2 μg/mL propidium iodide (PI), determined via flow cytometry. For all treatments, the % of cells is presented that: only have permeabilized membranes (gray), both have permeabilized membranes and HsLin06_18-FITC associated to their surface or internalized (orange) or only HsLin06_18-FITC associated to their surface or internalized (black). Data are means ± SEM in presented for *n* = 3 independent experiments. To analyse significant differences in the size of the subpopulations between the t0 and other time points, one-way ANOVA followed by Dunnett multiple comparison was performed, with brackets (in the color of the corresponding subpopulation) representing significance for that subpopulation. Only the primary time point that is significantly different from t0 is presented, with ^*^representing *P* < 0.05.

To study the relationship between HsLin06_18 internalization and membrane permeabilization in more detail, we monitored the kinetics of treated planktonic *C. albicans* cells every 10 min via flow cytometry (Figure 4 and Supplementary Figure S1). Our flow cytometric data revealed 4 subpopulations: (i) intact cells with HsLin06_18_FITC associated to their surface or internalized (HsL-F) (PI-/HsL-F+; black curve), (ii) cells with compromised membranes, but without HsLin06_18_FITC associated to their surface or internalized (PI+/HsL-F-/; gray curve), (iii) cells with compromised membranes and HsLin06_18_FITC associated to their surface or internalized (PI+/HsL-F+; orange curve) and (iv) intact cells without HsLin06_18_FITC associated to their surface or internalized (PI-/HsL-F-; not presented) (Figure 4). Only for the *C. albicans* cells that received the [caspofungin + HsLin06_18-FITC] combination, the population of cells that internalized or accumulated the peptide at the cell surface and had compromised membranes was significantly increased after 20 min (*P* = 0.0168) and this population was increasing over time (Figure 4C). This treatment also resulted in a significantly increased population of cells with only compromised membranes (*P* = 0.0182). However, over time, this population (approximately 13%) remained constant. In the case of treatment with HsLin06_18-FITC alone, a (small) population of cells (approximately 5%) appeared after 30 min of incubation that had only internalized or accumulated the peptide at the cell surface (*P* = 0.0482; Figure 4A). In the case of treatment with caspofungin alone, the population of cells with compromised membranes was not significantly increased after 40 min (Figure 4B). Overall, these data indicate that membrane permeabilization occurs only by the [caspofungin + HsLin06_18-FITC] combination and this event appears simultaneously with HsLin06_18-FITC internalization.

In addition, we determined the caspofungin dose that results in a similar fungicidal activity as that of the [caspofungin (0.01 μM) + HsLin06_18-FITC (4.6 μM)] combination. We found that upon 40 min of treatment, 80% of the population treated with the combination was dead as determined via plating assays (Supplementary Figure S2A), whereas concentrations up to 50 μM of caspofungin alone only resulted in 30% cell death (Supplementary Figure S2B). These data point to superior and fast fungicidal activity of the [caspofungin (0.01 μM) + HsLin06_18-FITC (4.6 μM)] combination against *C. albicans* planktonic cultures as compared to caspofungin alone, even when caspofungin is applied at 5,000-fold higher doses.

### The [caspofungin + HsLin06_18] combination affects biofilms of various non-*albicans Candida* species and a resistant *C. albicans* mutant

**Figure 5:**
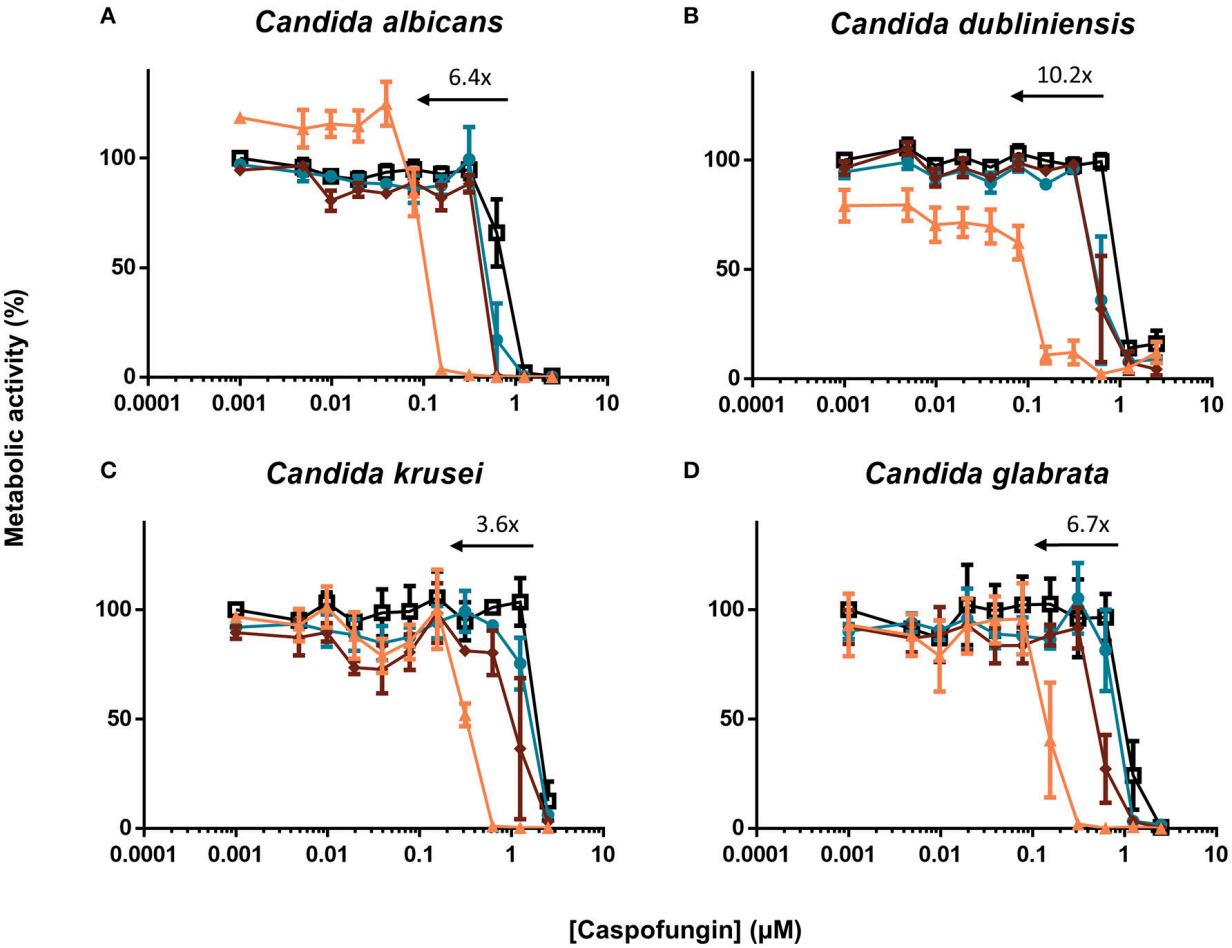
The [caspofungin + HsLin06_18] affects **(A)** *Candida albicans*, **(B)** *Candida dubliniensis*, **(C)** *Candida krusei* and **(D)** *Candida glabrata* biofilms in an *in vitro* microtiter plate assay, using XTT quantification. Dose-response curves of caspofungin are presented, with different colors for different HsLin06_18 concentrations; orange, 1.25 μM, red, 0.313 μM, blue, 0.156 μM, and black, 0 μM HsLin06_18. Black arrows represent the HsLin06_18's potentiation effect on caspofungin's action, i.e., the shift of the caspofungin concentration needed for 50% reduction of metabolic activity between the caspofungin and the [caspofungin + 1.25 μM HsLin06_18] treatment. Data are means ± SEM for *n* = 3 independent experiments.

Fungal infections caused by non-*albicans Candida* species are rising (Deorukhkar et al., 2014; Udayalaxmi et al., 2014; Muadcheingka and Tantivitayakul, 2015; Gong et al., 2016). Therefore, we tested the [caspofungin + HsLin06_18] combination against biofilm formation of *C. dubliniensis, C. krusei* and *C. glabrata*. We found that HsLin06_18 significantly increased the antibiofilm activity of caspofungin against all tested *Candida* species (Figure 5 and Supplementary Table S3). Similar to *C. albicans*, the BIC_50_ of caspofungin was reduced by approximately 5-10-fold in combination with HsLin06_18 against all tested *Candida* species. Together, these data point to a broad-spectrum superior antibiofilm activity of the [caspofungin + HsLin06_18] combination.

Echinocandins are the recommended drug to treat invasive fungal infections (Pappas et al., 2009). However, in many settings, such as prolonged and/or repeated drug exposure, fungal cells can acquire resistance resulting in therapeutic failure (Perlin, 2015). Using a caspofungin-resistant strain with mutations in Fks1, the caspofungin target, we tested the activity of the [caspofungin + HsLin06_18] combination. Although the BIC_50_ value of caspofungin was increased in the *fks1* mutant, HsLin06_18 could still increase the antibiofilm activity of caspofungin in a synergistic way (FICI values for 5 μM and 2.5 μM HsLin06_18 < 0.5) (Supplementary Table S4). These data indicate that HsLin06_18 acts synergistically with caspofungin against biofilms of caspofungin-resistant *C. albicans* mutants.

Using flow cytometry (Supplementary Figure S3), we found that most [caspofungin + HsLin06_18-FITC]-treated *fks1* mutant cells both had internalized the peptide and showed compromised membranes (approximately 40%), which is in line with the WT data (Figure 4C). Moreover, also a small but substantial subpopulation of cells that only internalized HsLin06_18-FITC or accumulated the peptide at the cell surface was present in the *fks1* mutants (approximately 8%), while few cells only had permeabilized membranes (approximately 0.4%) (Supplementary Figure S3). In contrast to *C. albicans* WT, the caspofungin dose (0.5 μM) alone did not induce membrane permeabilization of the resistant *fks1* mutant, as <0.2% of the *fks1* mutant cells had permeabilized membranes (Supplementary Figure S3). These results indicate that [caspofungin + HsLin06_18] acts in a similar way against *C. albicans* WT and caspofungin-resistant strains.

### The [caspofungin + HsLin06_18] combination affects biofilms in an *in vitro* catheter assay

**Figure 6:**
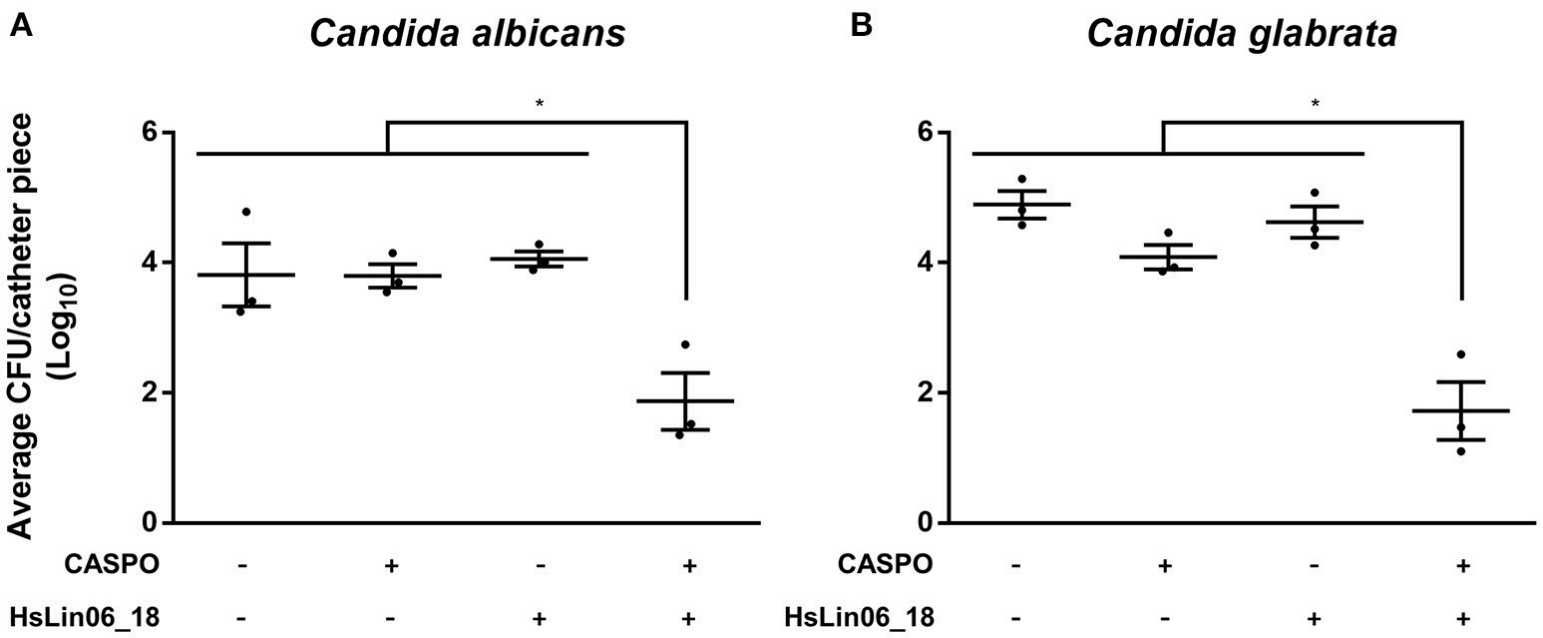
The [caspofungin (CASPO) + HsLin06_18] affects *Candida albicans* **(A)** and *C. glabrata* **(B)** biofilms in an *in vitro* catheter assay. The amount of the viable biofilm cells on the catheters treated with 0.4 μM CASPO, 0.5 μM HsLin06_18, its combination or the control (0.5% DMSO) was determined via CFU counting. Horizontal lines indicate the median values for log_10_ numbers of CFU ± SEM obtained per catheter piece, for *n* = 3 independent experiments. Significant differences between all treatments were determined via one-way ANOVA followed by Tukey multiple comparison, with ^*^representing *P* < 0.05.

As biofilms are often formed in or on medical devices (Cauda, 2009), we examined the activity of the [caspofungin + HsLin06_18] combination against *C. albicans* biofilm formation on serum pre-incubated catheters. To this end, *C. albicans* biofilms, grown on a 1 cm catheter piece, were treated with either caspofungin, HsLin06_18, [caspofungin + HsLin06_18] or 0.5% DMSO (control treatment), after which the amount of viable biofilm cells was determined via plating assays. First, the sub-lethal doses of caspofungin and HsLin06_18 in this setup were identified as 0.4 μM (Supplementary Figure S4) and 0.5 μM (Figure 6A), respectively. Two micromolar of HsLin06_18 already reduced the amount of viable biofilm cells on the catheters by 8-fold. Next, we found that the [caspofungin (0.4 μM) + HsLin06_18 (0.5 μM)] combination resulted in a significant (>98-fold) reduction of viable biofilm cells, compared to the control and mono treatments (Figure 6A). In addition, the [caspofungin + HsLin06_18] combination also significantly reduced the amount of viable *C. glabrata* biofilm cells by >99-fold, compared to the control or mono treatments (Figure 6B). Together, these data confirm the superior activity of the [caspofungin + HsLin06_18] combination against *Candida* biofilms in a setup mimicking *in vivo* conditions as serum pre-incubated catheter pieces were used.

### The [caspofungin + HsLin06_18] combination does not affect human hepatoma cells

We first investigated potential toxicity of HsLin06_18 on human HepG2 cells. HsLin06_18 was not cytotoxic up to 50 μM (which is 100-fold higher than used in the *in vitro* catheter assay) (Supplementary Figure S5). Next, cytotoxicity of the [caspofungin + HsLin06_18] combination was tested against HepG2 cells. The EC_50_, i.e., the half maximal effective concentration, of HsLin06_18 (EC_50_ > 50 μM) was not altered by the presence of caspofungin (up to 41.2 μM, which is 100-fold higher than used in the *in vitro* catheter assay). These results demonstrate non-toxicity of HsLin06_18 on these human cells, even in combination with caspofungin.

### The [caspofungin + HsLin06_18] combination reduces *Candida albicans* biofilms in an *in vivo* setup

We first tested the fungicidal activity of the [caspofungin + HsLin06_18] combination in serum. In contrast to RPMI media, in which the combination reduced cell survival by 80% as compared to the control or mono treatments (Supplementary Figure S2), the combination did not reduce cell survival in serum (data not shown). As HsLin06_18 might be degraded in serum, HsLin06_18 was further studied in terms of peptide stability. To this end, two strategies were investigated being cyclization of the peptide and the use of unnatural amino acids, as such types of peptides are known to be less prone to degradation. Cyclic HsLin06_18 was produced by forming one disulphide bridge between two cysteine amino acids, marked in gray in Table 2. From all three possible combinations of cysteines, HsLin06_18_21 could not be produced as the cysteine residues were too nearby to form a proper disulphide bond. The other cyclic peptides HsLin06_18_20 and HsLin06_18_22 were produced and screened for synergistic activity with caspofungin. Unfortunately, HsLin06_18_20 nor 22 could improve caspofungin's antibiofilm activity (Table 2), indicating that cyclization is not a good strategy to improve HsLin06_18's stability. Next, we replaced all L-amino acids of HsLin06_18 by D-amino acids, while in a another peptide (i.e., retro-inverso peptide) both the L-amino acids were replaced by D-amino acids and the order of the amino acids was reversed. Both peptides strongly inhibited *C. albicans* biofilm formation (BIC_50_(HsLin06_18_23) = 0.4 μM and BIC_50_(HsLin06_18_24) = 0.7 μM) when administered alone, while they did not act synergistically with caspofungin (data not shown).

**Figure 7:**
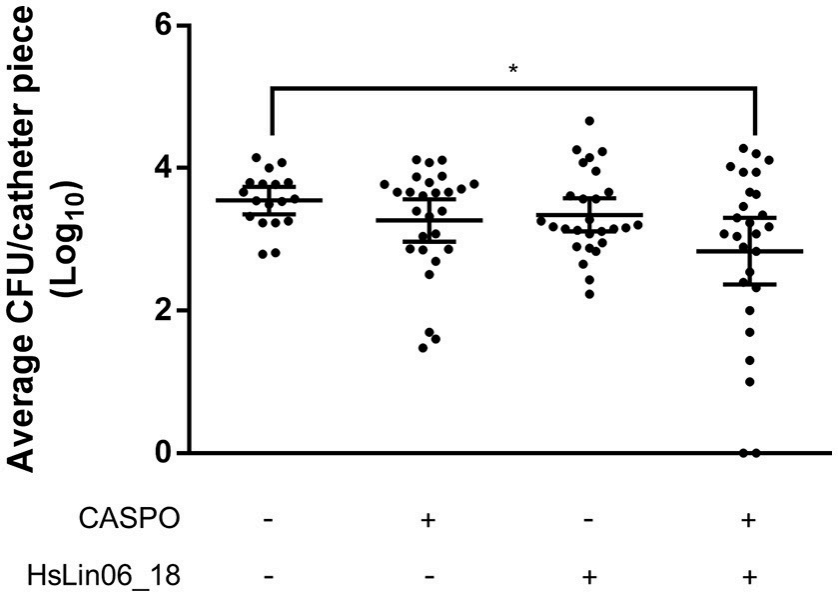
Effect of intravenous administration of caspofungin (CASPO) and HsLin06_18 on *Candida albicans* biofilm formation inhibition, using catheter pieces in a subcutaneous rat catheter model. Rats, containing nine catheter pieces each, were treated with 0.25 mg/kg/day CASPO, 2.5 mg/kg/day HsLin06_18, its combination or the control (0.9% NaCl and 0.2% DMSO) for 7 days, after which survival of viable biofilm cells on the catheters was determined via CFU counting. Horizontal lines indicate the median values for log_10_ numbers of CFU and 95% CI obtained per catheter piece, for *n* = 27 catheter pieces. Significant differences between all treatments were determined via one-way ANOVA followed by Tukey multiple comparison, with ^*^representing *P* < 0.05.

In conclusion, improving HsLin06_18's stability either via cyclization or via introduction of unnatural amino acids could not be obtained without affecting its activity. Hence, we proceeded with the *in vivo* studies with unmodified linear HsLin06_18 and assessed whether the [caspofungin + HsLin06_18] combination exhibits *in vivo* efficacy using a subcutaneous rat catheter model. First, the sub-lethal dose of (intravenously (IV) administered) caspofungin in this setup was identified as 0.25 mg/kg/day (Supplementary Figure S6). For the combination treatment, this sub-lethal caspofungin dose was supplemented with 2.5 mg/kg/day HsLin06_18 (IV administered). We found that *C. albicans* biofilm formation on catheter pieces in rats receiving the [caspofungin + HsLin06_18] combination was significantly reduced as compared to rats receiving the control (Figure 7) and reduced (although not significantly) as compared to rats receiving mono treatments (*P* = 0.2916 and *P* = 0.1413 for the caspofungin and the HsLin06_18 mono treatment, respectively). In a second *in vivo* experiment, we increased the HsLin06_18 dose to 10 mg/kg/day (IV administered) but this did not result in increased antibiofilm activity of the combination as compared to mono treatments (data not shown). As serum seems to inactivate the peptide *in vitro*, potential peptide degradation by serum components *in vivo* was circumvented in a third *in vivo* experiment by subcutaneous instead of intravenous administration of the peptide. In addition, we also increased the IV caspofungin dose to 0.5 mg/kg/day. In this experiment, *C. albicans* biofilm formation on catheter pieces in rats receiving the [caspofungin + HsLin06_18] combination was reduced (although not significantly) as compared to catheter pieces in rats receiving mono treatments (*P* = 0.1822 and *P* = 0.1925 for the caspofungin and the HsLin06_18 mono treatment, respectively) (Supplementary Figure S7). However, only in rats receiving the [caspofungin + HsLin06_18] combination, we observed sterility of some of the catheter fragments. In conclusion, the [caspofungin + HsLin06_18] combination reduces *C. albicans* biofilm formation *in vivo*, but the activity of the peptide seems reduced in these conditions.

## Discussion

Only three main classes of antimycotics (azoles, echinocandins, and polyenes) are currently used to treat fungal infections. Only the echinocandins and liposomal formulations of the polyene amphotericin B are effective against fungal biofilm-related infections (Fiori et al., 2011; Uppuluri et al., 2011). Hence, novel approaches to treat fungal biofilms are needed. The approach used in this study is a combination treatment, in which two compounds with different mode of actions are administrated simultaneously, thereby aiming at reducing potential cytotoxic side effects while increasing effectivity (Thevissen, 2016). In this study, we investigated the combination of caspofungin and linear HsAFP1-derived peptides (HsLins). Plant defensins, such as HsAFP1, are characterized by broad-spectrum antifungal activity (Osborn et al., 1995), *in vivo* efficacy (Tavares et al., 2008), and low *in vitro* frequency of resistance occurrence (Thevissen et al., 2007). Moreover, we recently discovered that HsAFP1 and HsLin06, the C-terminal part of HsAFP1, possess antibiofilm activity as well as synergistic activity with caspofungin (Vriens et al., 2015b). In this study, we found that both activities are uncoupled and further focussed on HsLin06-derived peptides with superior antibiofilm activity in combination with caspofungin. We identified HsLin06_18 as the smallest peptide that can reduce the BIC_50_ of caspofungin against *C. albicans* biofilms at least 10-fold and acts synergistically with caspofungin. Replacing one of the amino acids of HsLin06_18 by alanine did not change its superior antibiofilm activity in combination with caspofungin, suggesting that the exact amino acid sequence is not very important for this activity. The superior antibiofilm activity of the [caspofungin + HsLin06_18] combination is not limited to *C. albicans*, as it was also found against *C. dubliniensis, C. krusei* and *C. glabrata*. This points to broad-spectrum antibiofilm applications of the combination of caspofungin and HsLin06_18. Moreover, HsLin06_18's also increased the antibiofilm activity of other echinocandins, such as anidulafungin. Echinocandin potentiation has been demonstrated before by other peptides, such as colistin and DsS3 (1-16). Zeidler and colleagues suggested that echinocandins can weaken fungal cell walls, which can lead to enhanced fungal membrane targeting of these peptides (Zeidler et al., 2013). In line, Harris and Coote demonstrated enhanced DsS3 (1-16) uptake when the peptide was combined with caspofungin or anidulafungin (Harris and Coote, 2010).

We recently demonstrated that native HsAFP1 is accumulating at *S. cerevisiae*'s cell surface before being internalized, which is immediately followed by membrane permeabilization (Cools et al., submitted). Here, we showed that only in the presence of sub-inhibitory caspofungin doses, HsLin06_18 was internalized and caused membrane permeabilization of *C. albicans* cells. We suggest that caspofungin weakens fungal cell walls via inhibition of β-1,3-glucan synthase, as was suggested by Zeidler and colleagues (Zeidler et al., 2013), thereby increasing HsLin06_18's binding to fungal membranes and subsequently promoting the peptide's uptake. We also showed peptide internalization and membrane permeabilization for caspofungin-resistant mutants treated with the [caspofungin + HsLin06_18] combination, pointing to its broad activity spectrum. Moreover, as we showed the presence of a subpopulation of *fks1* mutant cells that only internalized the peptide, we suggest that, in line with HsAFP1 (Cools et al., submitted), HsLin06_18's internalization is occurring prior to membrane permeabilization.

Badrane and colleagues recently observed that moderate caspofungin doses rapidly result in elevated phosphatidylinositol(4,5)bisphosphate [PI(4,5)P_2_] levels as well as in the activation of the cell wall integrity (CWI) pathway in *C. albicans* (Badrane et al., 2016). As the native plant defensin HsAFP1 is known to interact with various phospholipids, including PI(4,5)P_2_ (Cools et al., submitted), increasing the levels of these phospholipids by moderate caspofungin doses (both in WT but also in *fks1* mutant strains) might result in increased, potentially PIP-dependent, peptide internalization. Our preliminary results using Differential Scanning Calorimetry (as described in Cools et al., submitted) show indeed that HsLin06_18 can interact with lipid vesicles containing PI(4,5)P_2_ (data not shown), supporting this hypothesis. However, additional experiments addressing HsLin06_18-PI(4,5)P_2_ interactions *in vivo* need to be performed in follow-up studies.

To mimic clinically relevant conditions (Ricicova et al., 2010), we also tested the activity of the combination against *C. albicans* biofilms grown on medical devices, such as catheter pieces. We observed that the combination of HsLin06_18 and caspofungin significantly reduced *C. albicans* biofilm formation *in vitro* as compared to the control or mono treatments. Next, we investigated the effect of the combination in a subcutaneous rat catheter model, which is a relevant *in vivo* model for investigating fungal biofilm-related infections (Ricicova et al., 2010). We found significantly reduced *C. albicans* biofilm formation on the catheters in rats receiving the combination treatment consisting of sub-inhibitory doses of caspofungin (0.25 mg/kg/day) and HsLin06_18 (2.5 mg/kg/day) as compared to rats receiving the control treatment. As we demonstrated that caspofungin, HsLin06_18 or its combination are not toxic for human (hepatoma) cells, higher HsLin06_18 doses (10 mg/kg/day) were used in a second experiment. However, increasing the HsLin06_18 dose did not result in increased antibiofilm activity of the [caspofungin + HsLin06_18] combination *in vivo*. Using another HsLin06_18 route of administration in rats did also not result in improved antibiofilm activity of the combination, indicating that HsLin06_18 is probably not very stable under *in vivo* conditions. Unfortunately, cyclization of the peptide nor the use of unnatural amino acids could stabilize the peptide without affecting its activity. Whether HsLin06_18 can be stabilized without affecting its antibiofilm activity needs to be investigated further. As we previously showed that the plant defensin RsAFP2 is prophylactically effective in mice against candidiasis when administered systemically at 7 or 14 mg/kg/day (Tavares et al., 2008), stable plant defensin-derived peptides might be effective as well under *in vivo* conditions.

## Conclusions

Plant defensins, such as HsAFP1 and its variants, show potential to treat fungal biofilm-related infections. In mono treatment, we earlier demonstrated that HsAFP1 and its variants can inhibit *C. albicans* biofilm formation on polystyrene and titanium surfaces, without being toxic to human cells (Vriens et al., 2015b). In the present study we show that a combination treatment consisting of HsAFP1 (variants) and caspofungin is effective against fungal biofilm formation on polystyrene and catheter substrates *in vitro* and *in vivo*. Hence, this study clearly indicates that HsAFP1 (variants) have a great potential in broad-spectrum antifungal/antibiofilm treatments.

## Ethics statement

We used a subcutaneous biofilm model system that we described in detail in Ricicova et al. (2010). This model system is ideally suited to study biofilms in animals as several independent biofilms can be investigated in one animal. Here we wanted to test the effect of a peptide as antifungal drug on fungal biofilms. Biofilm structures in this model are very similar to what is observed in patients, so our method is relevant toward human biology.

The animal experiments are approved by the KU Leuven ethical committee under the number: P069/2016. Animals are housed according to the national guidelines operational in Flanders.

One control group and three experimental groups:

Animals are picked randomly before the surgery (implant of the infected catheters). The analysis of the catheters is performed independently but treatment groups are known.

One experimental unit is one single animal with three similarly treated animals in one cage.

Surgery and anesthesia: Five minutes before the surgery rats are weighted and anestetized with a cocktail of drugs containing ketamine and domitor (1 ml of anestetic cocktail contains 600 μl of ketamine (Ketamine 1,000, 100 mg/ml) and 400 μl of medetomidine (Domitor, 1 mg/ml). Anestetics are injected intraperitoneally (100 μl of anesthetic cocktail per 100 g body weight, resulting in a dose of 60 mg/kg ketamine and 0.4 mg/kg medetomidine). From this moment and during the surgery rats are kept in individual cages placed on a warm pad for clinical histopathology (Leica HII220) in order to avoid hypothermia. During the whole surgery, ophthalmic ointment is applied directly on eyes to avoid drying (Alcon® Duratears®). In this stage, animals are ready for subcutaneous implant of serum-coated polyurethane catheters challenged with *C. albicans* cells. The lower back area will be shaved with a trimmer and the skin will be disinfected with chlorhexidine alcohol (0.5%). Small incision (approximately 1 cm long) will be made with sterile surgical scissors and up to nine catheter fragments will be implanted inside the subcutaneous area. Incision will be closed with 3–4 surgical staplers (Precise TM, 3 M) and gently disinfected. Immediately after surgery the local anesthesia (2% xylocaine gel) is applied on the incision site. Afterwards, anesthesia is reversed with intraperitoneal injection of atipamezole (Antisedan, 1 mg/kg). In order to avoid pain from the surgery, rats will be given buprenorphine solution diluted to 0.03 mg/ml with sterile saline. After the surgery, rats will be kept individually in separate cages placed on the warm pad with an access to water and food. Rats are monitored every 30 min for signs of pain.

Catheter removal and animal euthanasia: Before catheter removal rats will be sacrificed by CO_2_ inhalation. the skin will be disinfected, and catheter fragments will be removed and washed with PBS (2x).

200 g Female Sprague Dawley rats were used (Janvier Labs).

Housing: animals are kept in a dedicate animal room where only animals in an experiment are housed (no breeding). We keep three rats in one cage. They are filtertopped cages. Room humidity and temperature are continuously monitored.

Bedding material: wood based product, dust free. Lignocel, JRS.

Light/night circle: light from 7 a.m. until 7 p.m.

Free access to food (*ad libitum*)

- Food: pellets from Ssniff.
- Autoclaved tap water.

Cage enrichment: wood blocks and cotton balls.

Temperature: between 22 and 24°C.

Humidity: around 40–65%.

## Author contributions

Experiments were designed by TC, JD, SK, PV, BC, and KT; and KT coordinated the study. *In vitro* biofilm assays in microtiter plates as well as on catheters were performed by TC. Linear peptides were synthesized by JD and the *in vivo* catheter experiments were performed by TC, CS, SK, CL, and PV. Confocal microscopy was performed by TC and PV. Liposome experiments were done by TC, MR, and CB. The manuscript was written by TC and KT; and revised by CS, JD, SK, CL, PV, MR, CB, and BC. All authors have read and approved the final manuscript.

### Conflict of interest statement

The authors declare that the research was conducted in the absence of any commercial or financial relationships that could be construed as a potential conflict of interest.
