# Supplementary material for: A Linear 19-Mer Plant Defensin-Derived Peptide Acts Synergistically with Caspofungin against Candida albicans Biofilms
Source: Front Microbiol. 2017 Oct 20;8:2051. doi: 10.3389/fmicb.2017.02051 (PMC5655031; doi:10.3389/fmicb.2017.02051)
Supplement: Supplementary file 1 [file DataSheet1.docx]

Supplementary Material

**A linear 19-mer plant defensin-derived peptide acts synergistically with caspofungin against *Candida albicans* biofilms**

**Tanne L. Cools, Caroline Struyfs, Jan Wouter Drijfhout, Soňa Kucharíková, Celia Lobo Romero, Patrick Van Dijck, Marcelo H. S. Ramada, Carlos Bloch Jr. , Bruno P.A. Cammue^*^ and Karin Thevissen**

*** Correspondence: Bruno P. A. Cammue:** [bruno.cammue@kuleuven.be](mailto:bruno.cammue@kuleuven.be)


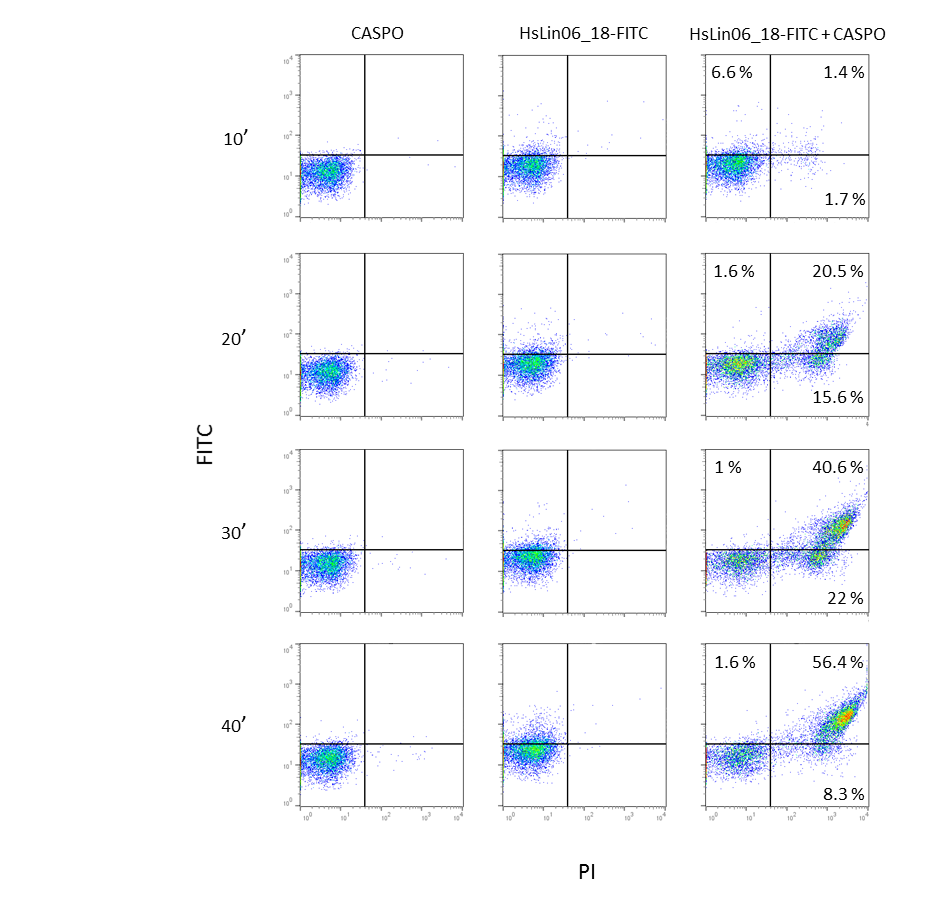


**Supplementary Figure S1: Kinetics of HsLin06_18-FITC internalization and membrane permeabilization from 1 representative experiment on planktonic Candida albicans cells treated with 0.01 µM caspofungin (CASPO), 4.6 µM HsLin06_18-FITC or [4.6 µM HsLin06_18-FITC + 0.01 µM CASPO] and 2 µg/mL propidium iodide (PI), determined via flow cytometry.** Percentages shown represent cells from 1 experiment that are FITC+/PI- (left upper corner), FITC+/PI+ (right upper corner) and FITC-/PI+ (right lower corner) respectively.


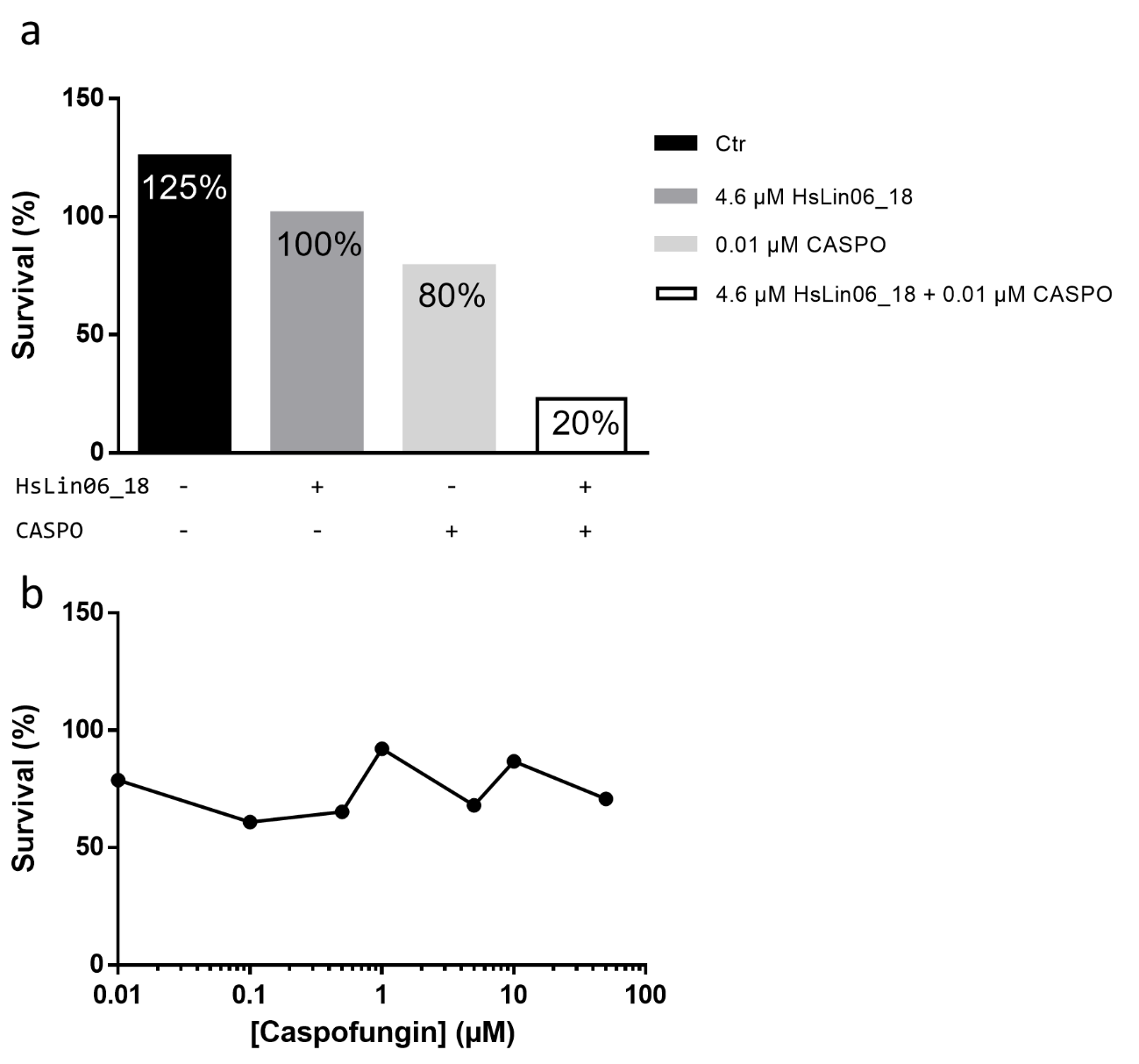


**Supplementary Figure S2: Cell death induced by (a) the control treatment, moderate caspofungin (CASPO) doses and/or HsLin06_18 or (b) high caspofungin doses on planktonic *Candida albicans* cultures.** (a) Survival of 40 minutes-treated cells was determined via CFU counting and results were expressed relative to t0. (b) The dose-response curve of caspofungin on the survival of *C. albicans* planktonic cells is presented. Results from one representative experiment out of 2 are shown.

 **Supplementary Figure S3: HsLin06_18-FITC uptake and membrane permeabilization in planktonic Candida albicans WT and fks1 mutant cells,** **determined via flow cytometry.** Cells were treated for 40 minutes with 0.5 µM caspofungin and/or 46 µM HsLin06_18-FITC (HsL-F) and 2 µg/mL propidium iodide (PI). Following subpopulations are presented: (i) cells that only have HsLin06_18-FITC associated to their surface or internalized (HsL-F uptake), (ii) both have permeabilized membranes and HsLin06_18-FITC associated to their surface or internalized (HsL-F uptake + membrane permeabilization) or (iii) only have permeabilized membranes (membrane permeabilization). Data are means ± SEM is presented for n = 3 independent experiments. To analyse significant differences in the size of the subpopulations between both strains, two-way ANOVA followed by Sidak multiple comparison was performed, with * representing P < 0.05.

**Supplementary Figure S4: Effect of caspofungin on Candida albicans biofilm formation inhibition in vitro on catheter pieces.** Survival of the viable biofilm-associated cells on the catheters was determined via CFU counting. Horizontal lines indicate the median values for log_10_ numbers of CFU ± SEM obtained per catheter piece, for n = 3 independent experiments. Significant differences between the control treatment and all caspofungin treatments were determined via one-way ANOVA followed by Dunnett multiple comparison, with * representing P < 0.05.


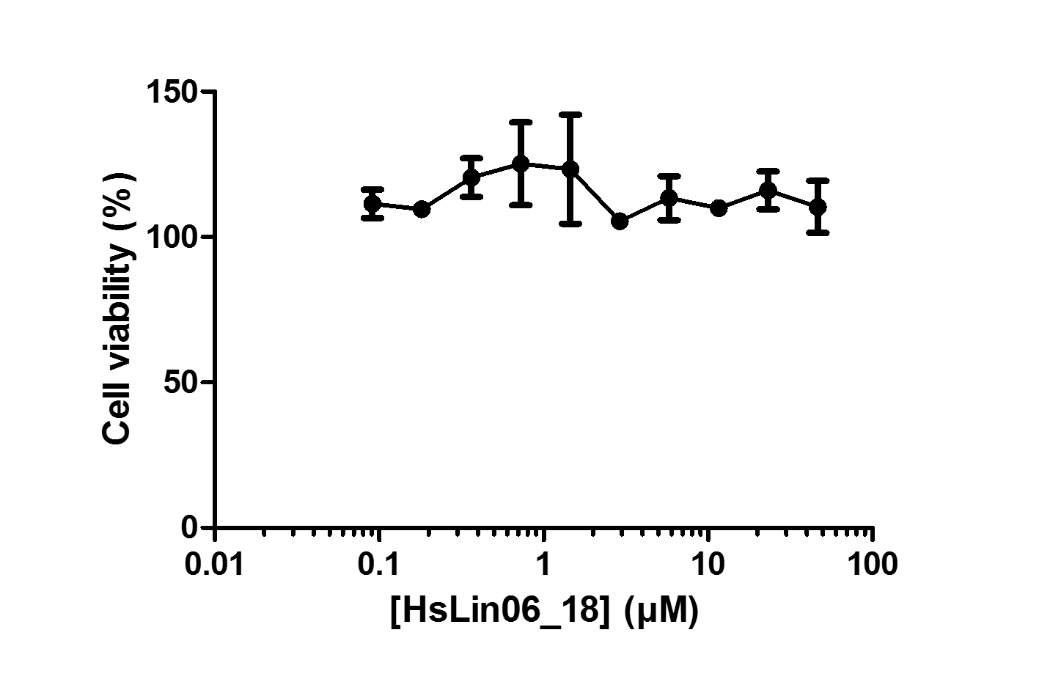


**Supplementary Figure S5: HsLin06_18 does not affect HepG2 cell viability.** HepG2 cells were treated with HsLin06_18 (0.09 µM – 50 µM) or control treatment (1% DMSO) for 24 h. Cell viability was determined by MTT staining and results were expressed relative to the control treatment. Data are means ± SEM of n = 4 independent experiments. Significant differences between all treatments were determined via one-way ANOVA followed by Tukey multiple comparison, with * representing P < 0.05.

**Supplementary Figure S6: Effect of intravenous administration of caspofungin on Candida albicans biofilm formation inhibition, using catheter pieces in a subcutaneous rat catheter model.** Rats, containing nine catheters each, were treated with 0-1 mg/kg/day caspofungin for 7 days, after which survival of viable biofilm-associated cells on the catheters was determined via CFU counting**.** Horizontal lines indicate the median values for log_10_ numbers of CFU and 95% CI obtained per catheter piece, for n = 18 catheter pieces. Significant differences between the control treatment and all caspofungin treatments were determined via one-way ANOVA followed by Dunnett multiple comparison, with * representing P < 0.05.


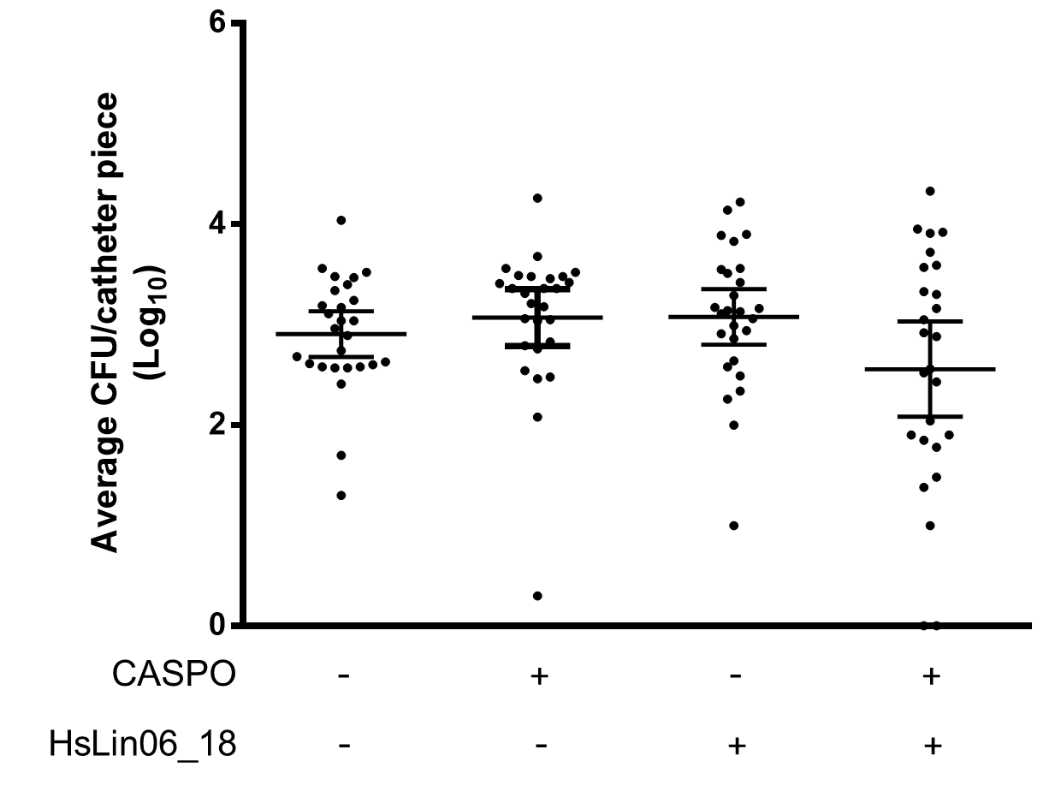


**Supplementary Figure S7: Effect of intravenous and subcutaneous administration of caspofungin (CASPO) and HsLin06_18, respectively, on Candida albicans biofilm formation inhibition, using catheter pieces in a subcutaneous rat catheter model.** Rats, containing nine catheter pieces each, were treated with caspofungin (0.5 mg/kg/day), HsLin06_18 (10 mg/kg/day), [caspofungin (0.5 mg/kg/day) + HsLin06_18 (10 mg/kg/day)] or control (Ctr; 0.9% NaCl and 0.2% DMSO) treatment for 7 days, after which survival of viable biofilm-associated cells on the catheters was determined via CFU counting**.** Horizontal lines indicate the median values for log_10_ numbers of CFU and 95% CI obtained per catheter piece, for n = 27 catheter pieces. Significant differences between all treatments were determined via one-way ANOVA followed by Tukey multiple comparison, with * representing P < 0.05.

**Supplementary Tables**

**Supplementary Table S1: FICI values of the combination treatment of HsLin06_18 with caspofungin on Candida albicans biofilms, in an in vitro microtiter plate assay ^b^**

| **Treatment** | **[HsLin06_18] (µM)** | **FICI**^a^ |
| --- | --- | --- |
| Caspofungin | 0 |  |
| Caspofungin + HsLin06_18 | 0.5 | **0.37** |
|  | 0.25 | **0.31** |
|  | 0.13 | 0.67 |
|  | 0.06 | 0.70 |
|  | 0.03 | 1.13 |
|  | 0.02 | 0.88 |

^a^ FICI, Fractional Inhibitory Concentration Index. FICI ≤ 0.5 indicates synergy between HsLin06_18 and caspofungin (marked in bold).

^b^ Data are means for n = 3 independent experiments.

**Supplementary Table S2: Potentiation activity of HsLin06_18 with different echinocandins: caspofungin, anidulafungin and micafungin on Candida albicans biofilms, in an in vitro microtiter plate assay**

| **Treatment** | **BIC50 (echinocandin) (µM) ± SEM** ^a^ | ***P*-value** ^b^ |
| --- | --- | --- |
| Caspofungin | 0.96 ± 0.040 |  |
| Caspofungin + 1.25 µM HsLin06_18 | 0.11 ± 0.046 | **0.0002** |
| Anidulafungin | 0.93 ± 0.120 |  |
| Anidulafungin + 1.25 µM HsLin06_18 | 0.38 ± 0.146 | **0.0449** |
| Micafungin | 0.40 ± 0.088 |  |
| Micafungin + 1.25 µM HsLin06_18 | 0.17 ± 0.040 | 0.0791 |

^a^ BIC50 values, i.e. the minimum inhibitory concentration resulting in 50% biofilm inhibition compared to the control treatment for n = 3 independent experiments.

^b^ Significant differences between the mono treatment with an echinocandin and the combination treatment with the echinocandin and HsLin06_18 were determined via an unpaired student t-test, with *P* < 0.05 as considered significantly different (marked in bold).

**Supplementary Table S3: Potentiation activity of HsLin06_18 with caspofungin (CASPO) on biofilms of various Candida strains, in an in vitro microtiter plate assay**

| **Strain** | **Treatment** | **BIC50 (CASPO) (µM) ± SEM** ^a^ | ***P*-value** ^b^ |
| --- | --- | --- | --- |
|  | CASPO | 0.70 ± 0.092 |  |
| *Candida albicans* | CASPO + 1.25 µM HsLin06_18 | 0.11 ± 0.009 | **0.0029** |
|  | CASPO | 0.92 ± 0.017 |  |
| *Candida dubliniensis* | CASPO + 1.25 µM HsLin06_18 | 0.09 ± 0.009 | **< 0.0001** |
|  | CASPO | 1.09 ± 0.012 |  |
| *Candida krusei* | CASPO + 1.25 µM HsLin06_18 | 0.30 ± 0.022 | **< 0.0001** |
|  | CASPO | 1.00 ± 0.115 |  |
| *Candida glabrata* | CASPO + 1.25 µM HsLin06_18 | 0.15 ± 0.033 | **0.0020** |

^a^ BIC50 values, i.e. the minimum inhibitory concentration resulting in 50% biofilm inhibition compared to the control treatment for n = 3 independent experiments.

^b^ Significant differences between the mono treatment with caspofungin and the combination treatment with caspofungin and HsLin06_18 were determined via an unpaired student t-test, with *P* < 0.05 as considered significantly different (marked in bold).

**Supplementary Table S4: FICI values of the combination treatment of HsLin06_18 with caspofungin (CASPO) on Candida albicans WT and fks1 mutant (mut), in an in vitro microtiter plate assay**

| **Strain** | **Treatment** | **[HsLin06_18] (µM)** | **BIC50 (CASPO) (µM) ± SEM** ^a^ | ***P*-value ^b^** | **FICI** ^c^ |
| --- | --- | --- | --- | --- | --- |
| WT | CASPO | 0 | 0.83 ± 0.096 |  |  |
|  | CASPO + HsLin06_18 | 5 | 0.09 ± 0.009 | **< 0.0001** | 1.10 |
|  |  | 2.5 | 0.10 ± 0.009 | **< 0.0001** | 0.61 |
|  |  | 1.25 | 0.11 ± 0.010 | **< 0.0001** | **0.38** |
|  |  | 0.63 | 0.13 ± 0.009 | **< 0.0001** | **0.28** |
|  |  | 0.31 | 0.30 ± 0.093 | **< 0.0001** | **0.40** |
|  |  | 0.16 | 0.43 ± 0.039 | **< 0.0001** | 0.54 |
| *fks1* mut | CASPO | 0 | 7.40 ± 1.361 |  |  |
|  | CASPO + HsLin06_18 | 5 | 1.5 ± 1.181 | **0.0001** | **0.31** |
|  |  | 2.5 | 2.58 ± 0.043 | **0.0014** | **0.40** |
|  |  | 1.25 | 3.68 ± 0.654 | **0.0158** | 0.52 |
|  |  | 0.63 | 4.01 ± 0.417 | **0.0308** | 0.55 |
|  |  | 0.31 | 4.81 ± 0.608 | 0.1376 | 0.66 |
|  |  | 0.16 | 4.93 ± 0.519 | 0.1680 | 0.67 |

^a^ BIC50 values, i.e. the minimum inhibitory concentration resulting in 50% biofilm inhibition compared to the control treatment for n = 3 independent experiments.

^b^ Significant differences between the mono treatment with caspofungin and the combination treatment with caspofungin and HsLin06_18 were determined via an unpaired student t-test, with *P* < 0.05 as considered significantly different (marked in bold).

^c^ FICI, Fractional Inhibitory Concentration Index. FICI ≤ 0.5 indicate synergy between HsLin06_18 and caspofungin (marked in bold).
